# The influence of immortal time bias in observational studies examining associations of antifibrotic therapy with survival in idiopathic pulmonary fibrosis: A simulation study

**DOI:** 10.3389/fmed.2023.1157706

**Published:** 2023-04-11

**Authors:** Qiang Zheng, Petr Otahal, Ingrid A. Cox, Barbara de Graaff, Julie A. Campbell, Hasnat Ahmad, E. Haydn Walters, Andrew J. Palmer

**Affiliations:** ^1^Menzies Institute for Medical Research, University of Tasmania, Hobart, TAS, Australia; ^2^NHMRC Centre of Research Excellence for Pulmonary Fibrosis, Camperdown, NSW, Australia; ^3^Department of Anaesthesiology (High–Tech Branch), First Affiliated Hospital of Anhui Medical University, Hefei, Anhui, China; ^4^Australian Government Department of Health and Aged Care, Tasmania (TAS) Office, Hobart, TAS, Australia; ^5^School of Medicine, University of Tasmania, Hobart, TAS, Australia

**Keywords:** immortal time bias, idiopathic pulmonary fibrosis, time–dependent, landmark, observational research

## Abstract

**Background:**

Immortal time bias (ITB) has been overlooked in idiopathic pulmonary fibrosis (IPF). We aimed to identify the presence of ITB in observational studies examining associations between antifibrotic therapy and survival in patients with IPF and illustrate how ITB may affect effect size estimates of those associations.

**Methods:**

Immortal time bias was identified in observational studies using the ITB Study Assessment Checklist. We used a simulation study to illustrate how ITB may affect effect size estimates of antifibrotic therapy on survival in patients with IPF based on four statistical techniques including time-fixed, exclusion, time-dependent and landmark methods.

**Results:**

Of the 16 included IPF studies, ITB was detected in 14 studies, while there were insufficient data for assessment in two others. Our simulation study showed that use of time–fixed [hazard ratio (HR) 0.55, 95% confidence interval (CI) 0.47–0.64] and exclusion methods (HR 0.79, 95% CI 0.67–0.92) overestimated the effectiveness of antifibrotic therapy on survival in simulated subjects with IPF, in comparison of the time–dependent method (HR 0.93, 95% CI 0.79–1.09). The influence of ITB was mitigated using the 1 year landmark method (HR 0.69, 95% CI 0.58–0.81), compared to the time–fixed method.

**Conclusion:**

The effectiveness of antifibrotic therapy on survival in IPF can be overestimated in observational studies, if ITB is mishandled. This study adds to the evidence for addressing the influence of ITB in IPF and provides several recommendations to minimize ITB. Identifying the presence of ITB should be routinely considered in future IPF studies, with the time–dependent method being an optimal approach to minimize ITB.

## Highlights

- **Question:** How immortal time bias (ITB) can affect effect size estimates of antifibrotic therapy on survival in patients with idiopathic pulmonary fibrosis (IPF)?
- **Findings:** The effectiveness of antifibrotic therapy on survival in patients with IPF can be overestimated in observational studies, if ITB is mishandled. Identifying the presence of ITB should be routinely considered in future IPF studies, with the time–dependent method being an optimal approach to minimize ITB.
- **Meaning:** Clinical decisions based on biased estimations may have potentially detrimental impact on clinical practice. This study adds to the evidence for addressing the influence of ITB in IPF studies and provides methods to ensure the true effect of treatments are estimated to ensure appropriate treatment for patients with IPF in future observational studies.

## Introduction

### Definition of immortal time bias

In the early 1970s, two observational studies (1, 2) examined associations between heart transplant and survival of patients and reported superior survival in treated patients compared to untreated controls. However, Gail (3) noted that there was an artificial survival advantage in the transplanted group, related to all treated patients needing to survive until time of treatment.

Immortal time refers to the waiting period before participants begin to receive interventions (e.g., antifibrotic therapy) since they will never become a treated patient if they die first or are censored (4, 5). However, no such period is allowed for the controls. When it is not adequately dealt with in analysis, a systematic error of immortal time bias (ITB) can occur, which distorts the real associations between interventions and outcomes in observational studies (6, 7).

### Susceptibility of idiopathic pulmonary fibrosis studies to ITB

Immortal time bias commonly occurs in cohort studies due to the nature of their observational settings. Idiopathic pulmonary fibrosis (IPF) studies examining associations between interventions and time–to–event outcomes are particularly susceptible to suffer from the influence of ITB due to two key reasons. First, identifying a specific commencement date for medication use may be difficult in available data sources, especially for individuals with a rapid disease trajectory, which may limit abilities to measure the immortal time. In practice, patients with IPF commonly experience a substantial duration of symptoms (such as cough and shortness of breath) before diagnosis. A previous study (8) found that the median time delay from symptom onset to diagnosis was 2.1 years, and 41% (*n* = 84) of incident patients with IPF reported initial misclassification of respiratory symptoms. Second, IPF is characterized by a high mortality risk, with a median survival time of about 3 years (9, 10). Observation periods in IPF studies are commonly short, and thereafter immortal person–time may account for a large proportion of the total observation period (person years) in the intervention group, which may induce substantial biases.

### Persistence of unaddressed ITB in IPF studies

The influence of ITB has been addressed in many disease areas such as diabetes (11), cancers (12), rheumatic diseases (13), orthopedics (14), and chronic obstructive pulmonary disease (COPD) (15), however, there is a paucity of data on the influence of ITB in IPF studies (16–19).

Several cohort studies (20, 21) have reported the protective effect of anti–gastric fluid reflux therapy on survival in IPF, while this survival benefit may have been affected by the influence of ITB (22, 23). In 2021, a meta–analysis (24) including 18 cohort studies reported the protective effect of antifibrotic therapy on survival of IPF; however, none were assessed or corrected for the influence of ITB. A recent commentary (25) has highlighted that the effectiveness of antifibrotic therapy in reducing the risk of death in IPF may be overestimated by the influence of ITB in a German cohort study (17).

### Current methods to mishandle immortal time in observational studies

Both time–fixed and exclusion methods are conventional but biased in IPF studies during analysis (26). The time–fixed method is defined as Cox proportional hazards regression models with a time–fixed definition for the study intervention, which can introduce misclassification bias by counting immortal person–time as part of the intervention group (11, 27). The exclusion method is defined as Cox models with a complete exclusion of immortal person–time from the analysis, which can introduce selection bias (11, 27).

### Appropriate methods to handle immortal time in observational studies

Both time–dependent and landmark methods are unbiased approaches and can be used to account for ITB (11, 28). The time–dependent method is defined as Cox models with a time–dependent definition for the study intervention. Any immortal person–time is added to the untreated comparator group for analysis (11). The landmark method is defined as Cox models with a landmark time, which excludes participants who have died or are censored before the landmark time (28). Participants are classified as exposed (intervention) group or unexposed (comparator) group based on their exposure status from cohort entry until the landmark time, and any newly exposed subjects during subsequent follow up after the landmark time are categorized into the comparator group (28). Although those two appropriate methods are gradually used in recent observational studies in the field of interstitial lung diseases (29, 30), there have been no studies in the field of IPF that evaluated effects of antifibrotic therapy with the incorporation of ITB.

### The objectives of our methodological study

With the above backdrop in mind, this methodological study aims to identify the presence of ITB in observational studies examining associations between antifibrotic therapy and survival in patients with IPF and illustrate how ITB may affect effect size estimates of those associations by assessment methods.

## Materials and methods

### Identification of ITB

A newly published ITB Study Assessment Checklist (4) was used to identify the presence of ITB in observational studies; this checklist includes five items: cohort entry, immortal time, intervention eligibility period, observation period, and statistical methods. From the IPF studies reviewed, cohort entry is defined as the time of IPF diagnosis if known or recruitment to a cohort if not. Immortal time is defined as the time between cohort entry and the initiation of antifibrotic therapy. Intervention eligibility period is defined as the duration of antifibrotic therapy for participants. Specific observation period for the intervention and comparator group is also needed to be provided. As mentioned previously, four statistical techniques include time-fixed, exclusion, time-dependent, and landmark methods.

We used the studies included in a recent meta–analysis (24) of survival benefit of antifibrotic therapy as examples to identify the presence of ITB. Two investigators (QZ and IC) independently assessed the presence of ITB and identified the statistical methods used in each observational study. If the intervention was a time–dependent exposure and there was an immortal time during the follow up, potential for ITB was deemed to exist. All discrepancies were discussed and resolved by consensus with a third investigator (AJP).

### A simulation study

To illustrate how ITB may affect the effectiveness of antifibrotic therapy on survival in IPF, we used a simulated dataset of subjects with IPF since access to the real world data was not available (22).

A simulation study is commonly used to estimate performance of statistical methods and illustrate how those methods can be utilized into practice (31). Individual survival data were simulated from a Weibull distribution with a proportional hazard function and censored at 5 years by using the “survsim package” in STATA (32). A hypothetic treatment variable (antifibrotic therapy) was generated from a binomial distribution with parameters *n* = 1,000 and *p* = 0.5. We incorporated the effect of antifibrotic therapy by defining a median background survival time of 3 years and a hazard ratio (HR) of death of 0.55, as estimated from a meta–analysis (24). For ITB illustration, we simulated that each subject with antifibrotic therapy had 1 year of immortal time.

Kaplan–Meier survival curves and Log-rank tests were used to compare 3 years survival between simulated subjects with and without antifibrotic therapy using the four statistical methods. In addition, Cox proportional hazards regression models were used to calculate crude HR [95% confidence interval (CI)] for mortality when ITB was considered. The time-dependent method was considered as the current gold-standard in this analysis (11, 28). We further quantified the difference in the effect estimates between the time-dependent method and other methods including time-fixed, exclusion, and landmark, as follows (33):


Difference=

$$\frac{\begin{array}{c} \left(\mathrm{HR}\,\mathrm{from}\,\mathrm{other}\,\mathrm{methods}\right)-\\ \left(\mathrm{HR}\,\mathrm{from}\,\mathrm{time}\,\mathrm{dependent}\,\mathrm{mothod}\right) \end{array}}{\left(\mathrm{HR}\,\mathrm{from}\,\mathrm{time}\,\mathrm{dependent}\,\mathrm{mothod}\right)}*100\%$$

Considering there are high heterogeneities on survival times or mortality outcomes in studies reporting IPF-related antifibrotic therapy (24), a sensitivity analysis was conducted to validate our estimates. We repeated the analyses defining a median background survival time of 2 years, and a HR of death of 0.38 in the unadjusted Cox model as estimated from a previous cohort study (19). For ITB illustration, we simulated that each subject with antifibrotic therapy had 1 year of immortal time.

All statistical analyses were conducted using STATA version 17.0 (34).

## Results

### Identification of ITB

There were 18 cohort studies (16–19, 35–48) in a recent meta–analysis (24), while one study (45) reporting white blood cell counts, and one study (46) reporting cross–sectional area of erector spinae muscle as the main intervention of interest were excluded from this study.

Of the 16 included studies (Table 1), 14 (16–19, 35, 37–39, 41–44, 47, 48) were the subject of ITB due to using time–dependent interventions (i.e., participants starting use of antifibrotic therapy at any time during the follow up period). Two studies (36, 40) were detected with uncertain status for ITB with obscure description of timelines for both intervention and comparator groups.

**TABLE 1:** Assessment of immortal time bias (ITB) in published studies reporting effects of antifibrotic therapy on survival of participants with idiopathic pulmonary fibrosis (IPF).

References	Country	ITB checklist*					Presence of ITB	Statistical methods
		**C1**	**C2**	**C3**	**C4**	**C5**		
Hosein et al. (40)	Canada	No	NA	NA	NA	No	NA	NA
Jo et al. (19)	Australia	Yes	Yes	No	Yes	No	Yes	Time-fixed
Margaritopoulos et al. (43)	Greece	Yes	Yes	No	Yes	No	Yes	Time-fixed
Zubairi et al. (48)	Pakistan	Yes	Yes	No	No	No	Yes	Time-fixed
Cerri et al. (36)	Italy	No	NA	NA	Yes	No	NA	NA
Dempsey et al. (16)	USA	Yes	Yes	No	Yes	No	Yes	Exclusion
Fernández-Fabrellas et al. (39)	Spain	Yes	Yes	No	No	No	Yes	Time-fixed
Kaunisto et al. (42)	Finland	Yes	Yes	No	Yes	No	Yes	Time-fixed
Zurkova et al. (48)	Czech Republic	Yes	Yes	No	Yes	No	Yes	Exclusion
Kang et al. (41)	South Korea	Yes	Yes	No	Yes	No	Yes	Exclusion
Adegunsoye et al. (18)	USA	Yes	Yes	No	Yes	No	Yes	Time-fixed
Alhamad et al. (35)	Saudi Arabia	Yes	Yes	No	Yes	No	Yes	Time-fixed
Behr et al. (17)	Germany	Yes	Yes	No	Yes	No	Yes	Exclusion
Dhooria et al. (37)	India	Yes	Yes	No	Yes	No	Yes	Time-fixed
Feng et al. (38)	China	Yes	Yes	No	Yes	No	Yes	Time-fixed
Moon et al. (44)	South Korea	Yes	Yes	No	Yes	No	Yes	Time-fixed

*The ITB Study Assessment Checklist; ITB: immortal time bias; IPF: idiopathic pulmonary fibrosis; NA: not applicable.

C1: Does study report cohort entry time–point for both intervention and comparator groups.

C2: Does immortal time exit in this study.

C3: Does study report intervention eligibility period for the intervention group.

C4: Does study report observation period for all groups.

C5: Does study report appropriate statistical methods (time–dependent or landmark methods) to address immortal time bias.

For statistical methods, the time–fixed method was used in ten studies (18, 19, 35, 37–39, 42–44, 47) and exclusion method was used in other four studies (16, 17, 41, 48). Specific statistical methods were not applicable in the remaining two studies (36, 40).

### A simulation study

#### Simulated subjects

Of the 1,000 simulated subjects with IPF, 483 were assigned to “take” antifibrotic therapy (antifibrotics users), and 517 did not receive antifibrotic therapy (non–users). The median (25th–75th percentiles) observation period from cohort entry to death was 3.5 (2.0, 5.0) years for the total population, 4.5 (2.7, 5.0) years for antifibrotics users, and 2.9 (1.5, 4.9) years for non–users, respectively.

#### Time-fixed method

**FIGURE 1:**
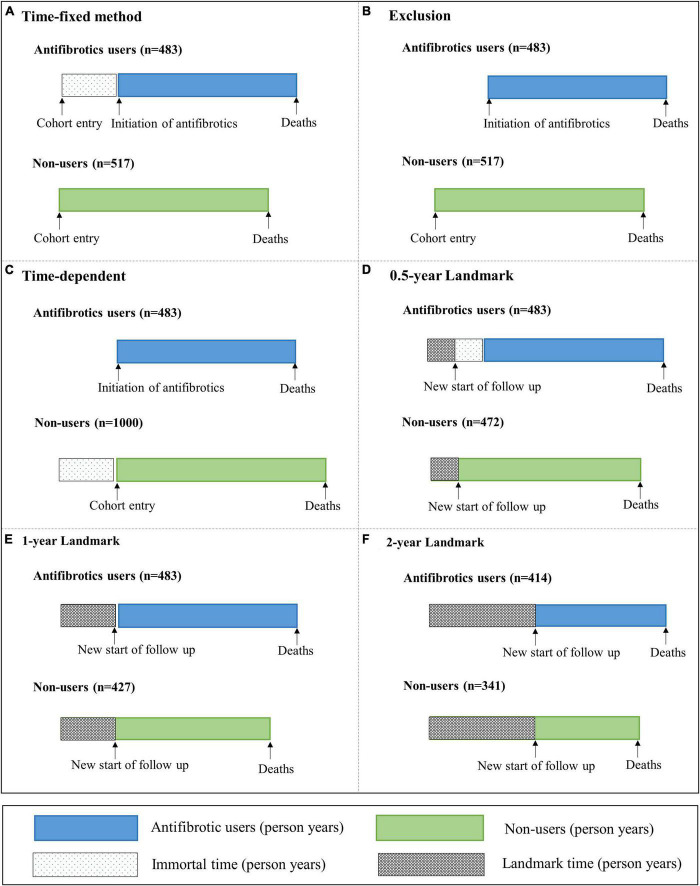
Illustration of allocating immortal time in four different methods. **(A)** Time–fixed method, **(B)** exclusion method, **(C)** time–dependent method, and **(D–F)** landmark method. For the time–fixed method, the immortal time was ignored and incorporated in the treated group. For the exclusion method, the immortal time was excluded from the study. For the time–dependent method, the immortal time was switched into the control group, with an additional 483 subjects being added into the control group. For the landmark method, 0.5, 1, and 2 years landmarks excluded 45, 90, and 245 simulated subjects who had died prior to this time point, respectively. Immortal time was defined as the time from cohort entry to the initiation of antifibrotic therapy. Landmark time was defined as a fixed time point, which was the same for all subjects.

**FIGURE 2:**
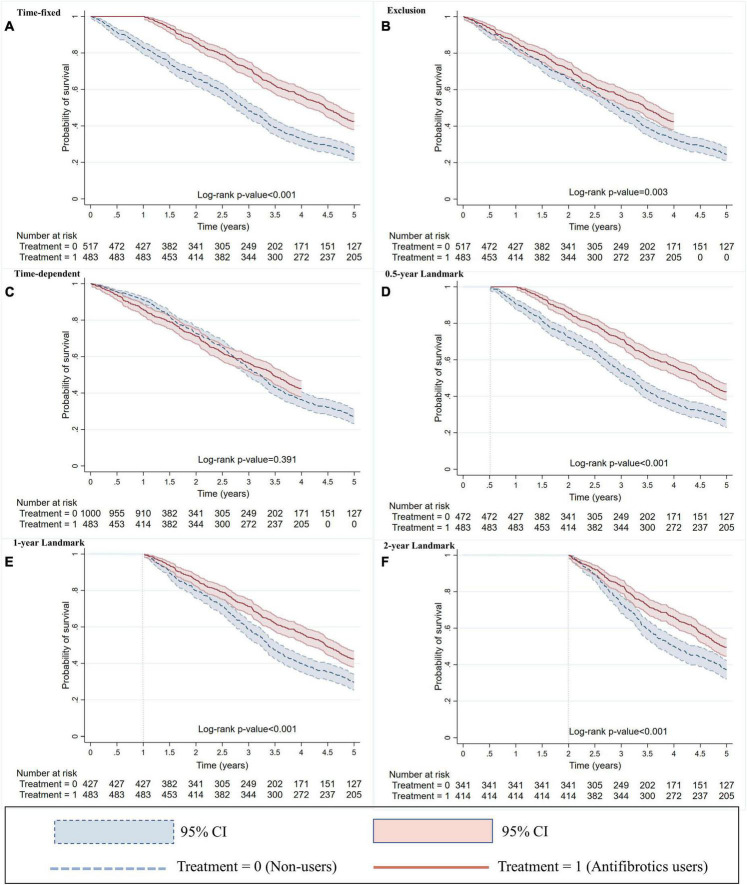
Kaplan–Meier survival curves with methods of **(A)** time–fixed, **(B)** exclusion, **(C)** time–dependent, and **(D–F)** landmark. For the time–fixed method, the immortal time was ignored and incorporated in the treated group. For the exclusion method, the immortal time was excluded from the study. For the time–dependent method, the immortal time was switched into the control group, with an additional 483 subjects being added into the control group. For the landmark method, 0.5, 1, and 2 years landmarks excluded 45, 90, and 245 simulated subjects who had died prior to this time point, respectively. Immortal time was defined as the time from cohort entry to the initiation of antifibrotic therapy. Landmark time was defined as a fixed time point, which was the same for all subjects.

Immortal time was 483 person years, which accounted for 26% of 1,855 person years for antifibrotics users in the time–fixed method. Immortal person–time were ignored and incorporated in the treated group (Figure 1A). The 3 years survival rate of antifibrotics users was significantly higher than non–users (71 vs. 48%; *P* < 0.001) (Figure 2A). Antifibrotics users had a significantly decreased risk of all–cause mortality compared to non–users using the time–fixed method (HR 0.55, 95% CI 0.47–0.64; *P* < 0.001) (Table 2).

**TABLE 2:** An illustration of the influence of immortal time bias (ITB) on associations between antifibrotic therapy and survival in simulated subjects with idiopathic pulmonary fibrosis (IPF) using four statistical methods.

	Antifibrotics users		Non–users				
	Person years*	**Deaths**	Person years*	**Deaths**	**Crude HR (95% CI)**	***P*-value**	**Difference (%)^#^**
**(a) Time–fixed method**							
Immortal person–time	483	0	0	0	–	–	–
At risk person–time	1,372	278	1,517	390	–	–	–
Total	1,855	278	1,517	390	0.55 (0.47, 0.64)	<0.001	–41
**(b) Exclusion method**							
Immortal person–time	0	0	0	0	–	–	–
At risk person–time	1,372	278	1,517	390	–	–	–
Total	1,372	278	1,517	390	0.79 (0.67, 0.92)	0.003	–15
**(c) Time–dependent method**							
Immortal person–time	0	0	483	0	–	–	–
At risk person–time	1,372	278	1,517	390	–	–	–
Total	1,372	278	2,000	390	0.93 (0.79,1.09)	0.391	0
**(d) 0.5 year landmark method**							
Immortal person–time	483	0	0	0	–	–	–
At risk person–time	1,372	278	1,504	345	–	–	–
Total	1,855	278	1,504	345	0.61 (0.52, 0.71)	<0.001	–34
**(e) 1 year landmark method**							
Immortal person–time	483	0	0	0	–	–	–
At risk person–time	1,358	281	1,469	300	–	–	–
Total	1,841	281	1,469	300	0.69 (0.58, 0.81)	<0.001	–26
**(f) 2 years landmark method**							
Immortal person–time	414	0	0	0	–	–	–
At risk person–time	1,335	209	1,339	214	–	–	–
Total	1,749	209	1,339	214	0.69 (0.57, 0.84)	<0.001	–26

Illustration models with methods of (a) time–fixed, (b) exclusion, (c) time–dependent, and (d)–(f) landmark. For the time–fixed method, the immortal time was ignored and incorporated in the treated group. For the exclusion method, the immortal time was excluded from the study. For the time–dependent method, the immortal time was switched into the control group, with an additional 483 subjects being added into the control group. For the landmark method, 0.5, 1, and 2 years landmarks excluded 45, 90, and 245 simulated subjects who had died prior to this time point, respectively. Immortal time was defined as the time from cohort entry to the initiation of antifibrotic therapy. Landmark time was defined as a fixed time point, which was the same for all subjects.

*Time from cohort entry until the occurrence of deaths.

^#^Difference in the effect estimates between the time-dependent methods and other methods.

ITB, immortal time bias; IPF, idiopathic pulmonary fibrosis; HR, hazard ratio; CI, confidence interval.

#### Exclusion method

All immortal person–time were excluded from the study in the exclusion method (Figure 1B). There was a significant difference in the 3 years survival rates between antifibrotics users and non–users using the exclusion method (56 vs. 48%; *P* = 0.003) (Figure 2B). Antifibrotics users had a significantly decreased risk of all–cause mortality compared to non–users using the exclusion method (HR 0.79, 95% CI 0.67–0.92; *P* = 0.003) (Table 2).

#### Time-dependent method

All immortal person–time were switched into the control group in the time–dependent method, with an additional 483 subjects being added into the control group (Figure 1C). The 3 years survival rates for antifibrotics users and non–users were similar (56 *versus* 53%; *P* = 0.391) (Figure 2C). There was no significant association between antifibrotic therapy and survival in subjects with IPF using the time-dependent method (HR 0.93, 95% CI 0.79–1.09; *P* = 0.391) (Table 2).

#### Landmark method

For the landmark method, 0.5, 1, and 2 years landmarks excluded 45, 90, and 245 simulated subjects who had died prior to this time point, respectively (Figures 1D–F). For the 0.5 year landmark method, the 3 years survival rate of antifibrotics users was significantly higher than non-users in subjects who survived more than 6 months (71 *versus* 53%; *P* < 0.001) (Figure 2D). For the 1 year landmark method, there was a significant difference in the 3 years survival rates between antifibrotics users and non–users in subjects who survived more than 1 year (71 *versus* 58%; *P* < 0.001) (Figure 2E). For the 2 years landmark method, the 3 years survival rate of antifibrotics users was significantly higher than non–users in subjects who survived more than 2 years (83 *versus* 73%; *P* < 0.001) (Figure 2F). Antifibrotics users were associated with a significantly decreased risk of all–cause mortality compared to non–users using 0.5 year landmark (HR 0.61, 95% CI 0.52–0.71; *P* < 0.001), 1 year landmark (HR 0.69, 95% CI 0.58–0.81; *P* < 0.001), and 2 years landmark (HR 0.69, 95% CI 0.57–0.84; *P* < 0.001), respectively (Table 2).

#### Difference in the effect estimates between four methods

Table 2 shows the difference in the effect estimates between the time-dependent method and other methods. Compared to the time-dependent method, use of time-fixed and exclusion methods overestimated the effectiveness of antifibrotic therapy in reducing the risk of all–cause mortality by 41 and 15%, respectively. For the 0.5, 1, and 2 years landmark methods, effectiveness of antifibrotic therapy was overestimated in reducing the risk of all–cause mortality by 34, 26 and 26%, respectively, compared to the time-dependent method.

#### Sensitivity analysis

After simulating data with a different background survival time and HR of death, our results remained consistent (Supplementary 1). Compared to the time-dependent method, use of time-fixed and exclusion methods overestimated the effectiveness of antifibrotic therapy in reducing the risk of all–cause mortality by 39 and 15%, respectively. For the 0.5, 1, and 2 years landmark methods, effectiveness of antifibrotic therapy was overestimated in reducing the risk of all–cause mortality by 32, 24 and 26%, respectively, compared to the time-dependent method.

## Discussion

### Main findings

To the best of our knowledge, this is one of few studies that highlights the importance of identifying and accounting for the influence of ITB in the field of IPF studies. We used the ITB Study Assessment Checklist to identify the presence of ITB in observational studies, and a simulation study (for the first time in the world) to illustrate how ITB can overestimate the survival impacts of IPF-related antifibrotic therapy in observational studies. Our findings have demonstrated the time–dependent method to be an optimal statistical approach to minimize ITB in IPF studies where immortal time is identified.

### The importance of addressing ITB in IPF studies

Observational studies examining effectiveness of medications on survival are highly susceptible to ITB in IPF due to substantial diagnostic delay and poor survival time. Further, time-fixed and exclusion methods are commonly used in IPF studies, which leads to overestimate the effectiveness of medications on survival in observational studies (22).

No examples of the assessment of ITB influence on survival of IPF using time–dependent or landmark methods have been published to date. Seven studies (11, 12, 28, 33, 49–51) were selected from a search for ITB literature based on PubMed, which were regarded as examples to illustrate how ITB can affect effect size estimates in other population. Detailed search strategies were provided in Supplementary 2. It has been demonstrated in previous studies that there could be substantial adjustments to the effect size estimates for interventions after correction for ITB (Table 3). ITB may also on some occasions lead to a reversal of the true effect estimate of interventions. For example, a previous study (52) reported that participants with type 2 diabetes using statins had a delay in disease progression (HR 0.74, 95% CI 0.58–0.95) compared to those without using stains; however, this association was reversed after correcting for ITB in the same dataset (HR 1.97, 95% CI 1.53–2.52) (11).

**TABLE 3:** Description of seven observational studies of the effects of interventions on study outcomes using various statistical methods for handling immortal time bias (ITB).

References	Country	Study size	Study subjects	Interventions*	Outcomes	Statistical methods	HR (95% CI)
Suissa (49)	Canada	3,524	COPD	Inhaled corticosteroids	All–cause mortality	Time–fixed	0.72 (0.58–0.88)^#^
						Time–dependent	0.94 (0.81–1.09)^#^
Shintani et al. (50)	USA	224	Mechanically ventilated patients	Delirium in the ICU	ICU length of stay	Time–fixed	1.90 (1.30–2.70)
						Time–dependent	1.10 (0.70–1.60)
Lévesque et al. (11)	Canada	11,661	Diabetes	Statins	Disease progression	Time–fixed	0.74 (0.58–0.95)
						Time–dependent	1.97 (1.53–2.52)
Mi et al. (28)	USA	52,741	COPD	Inhaled corticosteroids	3 years mortality	Time–fixed	0.55 (0.53–0.57)
						Exclusion	0.66 (0.64–0.69)
						Time–dependent	0.97 (0.93–1.00)
						3 months landmark	0.94 (0.90–0.97)
						6 months landmark	0.99 (0.95–1.03)
						9 months landmark	1.02 (0.97–1.06)
						12 months landmark	1.01 (0.97–1.07)
Weberpals et al. (12)	Germany	9,876	Prostate cancer	Beta–blockers	All–cause mortality	Time–fixed	0.68 (0.60–0.77)
						Time–dependent	1.13 (1.00–1.28)
			Colorectal caner			Time–fixed	0.51 (0.47–0.57)
						Time–dependent	1.15 (1.05–1.26)
			Lung caner			Time–fixed	0.42 (0.38–0.46)
						Time–dependent	1.04 (0.96–1.13)
			Pancreatic cancer			Time–fixed	0.34 (0.22–0.51)
						Time–dependent	1.10 (0.84–1.44)
Wallis et al. (33)	Canada	38,340	Men aged ≥ 66 years	Tertile 1; exposure of TRT ≤ 120 days	All–cause mortality	Time–fixed	1.23 (1.14–1.33)
						Time–dependent	1.11 (1.03–1.20)
				Tertile 2; exposure of TRT 121–510 days		Time–fixed	1.02 (0.95–1.11)
						Time–dependent	0.90 (0.83–0.97)
				Tertile 3; exposure of TRT ≥ 511 days		Time–fixed	0.56 (0.52–0.61)
						Time–dependent	0.67 (0.62–0.73)
Choi et al. (51)	Korea	16,769	Ulcerative colitis	5–Aminosalicylic acid	Incidence of colorectal cancer	Time–fixed	0.18 (0.09–0.35)
						6 months landmark	0.58 (0.35–0.97)
						1 year landmark	0.59 (0.32–1.09)
						2 years landmark	0.55 (0.25–1.19)

*“Intervention” might be an intervention, treatment, or exposure.

^#^Rate ratio; ITB, immortal time bias; COPD, chronic obstructive pulmonary fibrosis; ICU, intensive care unit; TRT, testosterone replacement; HR, hazard ratio; CI, confidence interval.

### Identification of ITB

Of the 16 studies reviewed, we found that 14 studies were detected with presence of ITB based on the ITB checklist, while there were insufficient data for assessment of two others. This is consistent with a recently published Letter to Editor (26) that summarized observational studies reporting the effectiveness of antifibrotic therapy on mortality of IPF and identified 14 studies (16–19, 35, 41, 42, 44, 45, 53–57) presenting ITB. Compared to a previous study (26), we have included more studies and have provided more detailed information by using the ITB checklist and conducting a simulation study to identify and account for ITB in IPF studies. Furthermore, Kaplan-Meier survival curves are plotted for illustrating correction of ITB, which could be used to identify the presence of ITB through observing the initial part of the survival curves when there is substantial variation in the slopes between antifibrotics users and non–users. The immediate marked separation of survival plots where present in previous IPF studies means that ITB was not taken into account (17, 19). In addition, all STATA code for data generation and modeling are given in Supplementary 3 which provide detailed information to repeat our analyses or validate our results in real datasets for future studies.

### Correction of ITB

Our simulation study showed that use of time–fixed and exclusion methods overestimated the effectiveness of antifibrotic therapy in reducing the risk of all–cause mortality by 41 and 15%, respectively, compared to the time–dependent method. This is consistent with the findings of a methodological study which examined the effectiveness of inhaled corticosteroids on survival in a real dataset of patients with COPD (28). By using various methods to handle ITB, the investigators found that both the time–fixed method (HR 0.55, 95% CI 0.53–0.57) and exclusion method (HR 0.66, 95% CI 0.64–0.69) considerably overestimated the effectiveness of inhaled corticosteroids in comparison of the time–dependent method (HR 0.97, 95% CI 0.93–1.00) which the authors identified as a “gold standard.”

The time–dependent method is closest to the true effect of interventions on study outcomes that was first reported in 1974 by Mantel and Byar (58). In the early 2000s, Suissa (49, 59, 60) comprehensively addressed the potential influence of ITB on associations between medications (such as inhaled corticosteroids and beta–agonists) and survival in COPD. However, such an optimal approach requires adequate information to calculate the immortal time.

The landmark method is an alternative approach to the time–dependent method that was introduced in 1983 by Anderson et al. (61). We found that this method can mitigate the influence of ITB compared to the time–fixed method, but its performance highly dependent on the timepoint chosen. Thus, multiple landmark time points are commonly set to account for ITB. A previous study (28) set four landmark time points (3, 6, 9, and 12 months) to examine the effectiveness of inhaled corticosteroids on survival in COPD, but only found a significant effect at a 3 months landmark model. It should be noted that subjects who have died are excluded prior to the landmark time, thus the effect of antifibrotic therapy should be interpreted as being among subjects who survive at least to the defined landmark time. In addition, use of landmark method has a key limitation: subjects are excluded from the analysis which reduces statistical power; this is particularly important in critical care research where events are usually more common early in the disease process in subjects with severe diseases such as IPF.

### Strengths and limitations

This study adds to the evidence for addressing the influence of ITB in IPF studies and provides several recommendations to minimize ITB in future observational studies. First, the ITB Study Assessment Checklist should be used to avoid ITB at the stage of study design and data analysis. f, the prevalent new-user design provides a comparison of exposed patients with time-matched unexposed controls during follow up, which might avoid ITB. (23) Third, a longer observation period can be used to mitigate the influence of ITB by reducing the proportion of immortal person–time in the total observation period (person years) in the intervention group. While this might be difficult in some patients with rapid disease progression. In addition, studies should collect and utilize adequate data to calculate immortal person–time. Lastly, the use of time–dependent and landmark methods can account for the influence of ITB in observational studies during data analysis.

Lack of validating our results in a real-world dataset is the main limitation for this study. Our simulation study can only confirm the direction of ITB and illustrate how it can overestimate the survival impacts of IPF-related antifibrotic therapy, while this study is limited to estimate the real magnitude of the effect size of this bias. In addition, the simulated data are generated based on a few reasonable assumptions in support of the modeling that illustrate the impact of ITB, while there are high heterogeneities on survival times and mortality outcomes for IPF-related antifibrotic therapy reported from a previous meta-analysis (24). However, results from our sensitivity analysis remained consistent after simulating datasets based on a different background of survival time and mortality outcome. Future studies should quantify the effect size estimates of the influence of ITB on associations between antifibrotic therapy and survival in patients with IPF based on data linkage with filling of prescriptions, although it could be challenging for getting the data required for such analyses.

## Conclusion

The effectiveness of antifibrotic therapy on survival in IPF is likely to be overestimated in observational studies, if ITB is not handled appropriately. Identifying the presence of ITB should therefore be routinely considered and reported in future IPF studies, and we recommend the use of time–dependent method to optimally account for the influence of ITB in observational studies. Clinical decisions based on biased estimations may have potentially detrimental impact on clinical practice. This study provides methods to ensure the true effect of treatments are estimated to ensure appropriate treatment for patients with IPF in future observational studies.

## Data availability statement

The original contributions presented in this study are included in the article/Supplementary material, further inquiries can be directed to the corresponding author.

## Ethics statement

Ethical review and approval was not required for the study on human participants in accordance with the local legislation and institutional requirements. Written informed consent for participation was not required for this study in accordance with the national legislation and the institutional requirements.

## Author contributions

QZ designed the study and wrote the first draft of the manuscript. PO contributed to the statistical review and manuscript writing. EW, IC, BG, JC, and HA contributed to the study design and manuscript writing. AP contributed to the conceptualization, study design, and manuscript writing. All authors contributed to the revisions and agreed to the final manuscript.
