# Supplementary material for: The influence of immortal time bias in observational studies examining associations of antifibrotic therapy with survival in idiopathic pulmonary fibrosis: A simulation study
Source: Front Med (Lausanne). 2023 Apr 11;10:1157706. doi: 10.3389/fmed.2023.1157706 (PMC10126672; doi:10.3389/fmed.2023.1157706)
Supplement: Supplementary file 1 [file Data_Sheet_1.DOCX]

**Supplementary Material**

**The influence of immortal time bias in observational studies examining associations of antifibrotic therapy with survival in idiopathic pulmonary fibrosis: a simulation study**

Qiang Zheng^1,2,3^, Petr Otahal^1^, Ingrid A. Cox^1,2^, Barbara de Graaff^1,2^, Julie A. Campbell^1^, Hasnat Ahmad^1,4^, E. Haydn Walters^1,2,5†^, Andrew J. Palmer^1,2†*^.

**AFFLIATIONS:**

^1^Menzies Institute for Medical Research, University of Tasmania, Hobart, Tasmania, Australia

^2^NHMRC Centre of Research Excellence for Pulmonary Fibrosis, Camperdown, NSW, Australia

^3^Department of Anaesthesiology (High–Tech Branch), the First Affiliated Hospital of Anhui Medical University, Hefei, Anhui, China

^4^Australian Government Department of Health and Aged Care, Tasmania (TAS) Office, Australia

^5^School of Medicine, University of Tasmania, Hobart, Tasmania, Australia

^†^ These authors share senior authorship

***Correspondence**: Andrew J. Palmer, Menzies Institute for Medical Research, Private Bag 23, Hobart, TAS 7001, Australia. Email: [andrew.palmer@utas.edu.au](mailto:andrew.palmer@utas.edu.au)

**Supplement 1: Sensitivity analysis**

**TABLE S1** An illustration of the influence of ITB on associations between antifibrotic therapy and survival in simulated subjects with IPF using four statistical methods

|  | **Antifibrotics users** | | **Non–users** | |  |  |  |
| --- | --- | --- | --- | --- | --- | --- | --- |
|  | **Person years ^*^** | **Deaths** | **Person years ^*^** | **Deaths** | **Crude**  **HR (95% CI)** | ***P* value** | **Difference (%) ^#^** |
| **a) Time–fixed method** | | | | | |  |  |
| Immortal person–time | 483 | 0 | 0 | 0 |  |  |  |
| At risk person–time | 1,513 | 210 | 1,517 | 390 |  |  |  |
| Total | 1,996 | 210 | 1,517 | 390 | 0.38 (0.32, 0.45) | <0.001 | -39 |
| **b) Exclusion method** | | | | | |  |  |
| Immortal person–time | 0 | 0 | 0 | 0 |  |  |  |
| At risk person–time | 1,513 | 210 | 1,517 | 390 |  |  |  |
| Total | 1,513 | 210 | 1,517 | 390 | 0.53 (0.45, 0.63) | <0.001 | -15 |
| **c) Time–dependent method** | | | | | |  |  |
| Immortal person–time | 0 | 0 | 483 | 0 |  |  |  |
| At risk person–time | 1,513 | 210 | 1,517 | 390 |  |  |  |
| Total | 1,513 | 210 | 2,000 | 390 | 0.62 (0.52,0.74) | <0.001 | 0 |
| **d) 0.5–year landmark method** | | | | | |  |  |
| Immortal person–time | 483 | 0 | 0 | 0 |  |  |  |
| At risk person–time | 1,372 | 210 | 1,504 | 345 |  |  |  |
| Total | 1,996 | 210 | 1,504 | 345 | 0.42 (0.35, 0.49) | <0.001 | -32 |
| **e) 1–year landmark method** | | | | | |  |  |
| Immortal person–time | 483 | 0 | 0 | 0 |  |  |  |
| At risk person–time | 1,513 | 210 | 1,469 | 300 |  |  |  |
| Total | 1,996 | 210 | 1,469 | 300 | 0.47 (0.39, 0.56) | <0.001 | -24 |
| **f) 2–year landmark method** | | | | | |  |  |
| Immortal person–time | 432 | 0 | 0 | 0 |  |  |  |
| At risk person–time | 1,485 | 159 | 1,339 | 214 |  |  |  |
| Total | 1,917 | 159 | 1,339 | 214 | 0.46 (0.37, 0.56) | <0.001 | -26 |

Illustration models with methods of a) Time–fixed, b) Exclusion, c) Time–dependent, and d) – f) Landmark. For the time–fixed method, the immortal time was ignored and incorporated in the treated group. For the exclusion method, the immortal time was excluded from the study. For the time–dependent method, the immortal time was switched into the control group, with an additional 483 subjects being added into the control group. For the landmark method, 0.5–year, 1–year, and 2–year landmarks excluded 45, 90, and 227 simulated subjects who had died prior to this time point, respectively. Immortal time was defined as the time from cohort entry to the initiation of antifibrotic therapy. Landmark time was defined as a fixed time point, which was the same for all subjects. ^*^: Time from cohort entry until the occurrence of deaths. ^#^: Difference in the effect estimates between the time-dependent methods and other methods. ITB, immortal time bias; IPF, idiopathic pulmonary fibrosis; HR, hazard ratio; CI, confidence interval.

**Supplement 2: Search strategy**

The idea of this manuscript is from a recent commentary (1) that was published in March 2021. This commentary has highlighted that the effectiveness of antifibrotic therapy in reducing the risk of death in participants with IPF may be overestimated by the influence of ITB in a German cohort study (2). Therefore, we started to conduct literature research in PubMed from 1^st^ January 1900 to 1^st^ May 2021.

There were three search strategies (Table S1). First, the search strategy involved several combinations of “idiopathic pulmonary fibrosis”, “antifibrotic therapy”, “immortal time bias” and their synonyms. No records were identified. Second, the search strategy involved several combinations of “idiopathic pulmonary fibrosis”, “immortal time bias” and their synonyms. Only one methodological study based on simulated subjects was identified (3). Lastly, the search strategy only involved “immortal time bias”. A total of 989 studies were identified. Considering no examples of the assessment of ITB influence on survival of IPF using time–dependent or landmark methods have been published to date, seven studies (4-10) were selected from a search for ITB literature. Those studies were regarded as examples to illustrate how ITB can affect effect sizes estimates in other population. ITB can affect effect size estimates in those population as well as IPF.

**TABLE S1** Search strategies for ITB literature in PubMed

| **Search strategy 1:**  (("idiopathic pulmonary fibrosis"[Text Word] OR "usual interstitial pneumonitis"[Text Word] OR "IPF"[Text Word]) AND ("antifibrotic therapy"[Text Word] OR "antifibrotics"[Text Word] OR "pirfenidone"[Text Word] OR "nintedanib"[Text Word]) AND ("ITB"[Text Word] OR "immortal time bias"[Text Word]) AND "humans"[MeSH Terms] AND 1900/01/01:2021/05/01[Date - Publication]) AND (humans[Filter]) **(N=0)** |
| --- |
| **Search strategy 2:**  (("idiopathic pulmonary fibrosis"[Text Word] OR "usual interstitial pneumonitis"[Text Word] OR "IPF"[Text Word]) AND ("ITB"[Text Word] OR "immortal time bias"[Text Word]) AND "humans"[MeSH Terms] AND 1900/01/01:2021/05/01[Date - Publication] AND "humans"[MeSH Terms]) AND (humans[Filter]) **(N=1)** |
| **Search strategy 3:**  (("ITB"[Text Word] OR "immortal time bias"[Text Word]) AND "humans"[MeSH Terms] AND 1900/01/01:2021/05/01[Date - Publication]) AND (humans[Filter]) **(N=989)** |

**Reference**

1. Suissa S, Assayag D. Mortality benefit with antifibrotics in idiopathic pulmonary fibrosis: real world evidence or bias? Eur Respir J 2021: 57(3).

2. Behr J, Prasse A, Wirtz H, Koschel D, Pittrow D, Held M, Klotsche J, Andreas S, Claussen M, Grohé C, Wilkens H, Hagmeyer L, Skowasch D, Meyer JF, Kirschner J, Gläser S, Kahn N, Welte T, Neurohr C, Schwaiblmair M, Bahmer T, Oqueka T, Frankenberger M, Kreuter M. Survival and course of lung function in the presence or absence of antifibrotic treatment in patients with idiopathic pulmonary fibrosis: long-term results of the INSIGHTS-IPF registry. European Respiratory Journal 2020: 56(2): 1902279.

3. Tran T, Suissa S. The Effect of Anti-Acid Therapy on Survival in Idiopathic Pulmonary Fibrosis: A methodological Review of Observational Studies. European Respiratory Journal (2018) 51(6):1800376. doi: 10.1183/13993003.00376-2018.

4. Lévesque LE, Hanley JA, Kezouh A, Suissa S. Problem of Immortal Time Bias in Cohort Studies: Example Using Statins for Preventing Progression of Diabetes. Bmj (2010) 340:b5087. Epub 2010/03/17. doi: 10.1136/bmj.b5087.

5. Weberpals J, Jansen L, Van Herk-Sukel MPP, Kuiper JG, Aarts MJ, Vissers PAJ, et al. Immortal Time Bias in Pharmacoepidemiological Studies on Cancer Patient Survival: Empirical Illustration for Beta-Blocker Use in Four Cancers with Different Prognosis. European Journal of Epidemiology (2017) 32(11):1019-31. doi: 10.1007/s10654-017-0304-5.

6. Mi X, Hammill BG, Curtis LH, Lai EC-C, Setoguchi S. Use of the Landmark Method to Address Immortal Person-Time Bias in Comparative Effectiveness Research: A Simulation Study. Statistics in Medicine (2016) 35(26):4824-36. doi: 10.1002/sim.7019.

7. Wallis CJD, Saskin R, Narod SA, Law C, Kulkarni GS, Seth A, et al. Estimating the Effect of Immortal-Time Bias in Urological Research: A Case Example of Testosterone-Replacement Therapy. BJU International (2017) 120(4):584-90. doi: 10.1111/bju.13918.

8. Suissa S. Inhaled Steroids and Mortality in Copd: Bias from Unaccounted Immortal Time. European Respiratory Journal (2004) 23(3):391-5. doi: 10.1183/09031936.04.00062504.

9. Shintani AK, Girard TD, Eden SK, Arbogast PG, Moons KGM, Ely EW. Immortal Time Bias in Critical Care Research: Application of Time-Varying Cox Regression for Observational Cohort Studies*. Critical Care Medicine (2009) 37(11):2939-45. doi: 10.1097/ccm.0b013e3181b7fbbb.

10. Choi YY, Lee JK, Kim H-S, Kim DW, Kim HM, Kang DR. Medications and the Risk of Colorectal Cancer in Patients with Inflammatory Bowel Diseases: Use of the Landmark Method. Yonsei Medical Journal (2021) 62(11):997. doi: 10.3349/ymj.2021.62.11.997.

**Supplement 3: STATA code**

//====================Simulating survival data=============

ssc install survsim

clear

set seed 12345

set obs 1000

generate Treatment = rbinomial(1,0.5)

survsim Stime, distribution(weibull) lambdas(0.2) gammas(1.2) covariates(Treatment -0.25)

gen Imtime=1 if Treatment==1

replace Stime=Stime+Imtime if Treatment==1

generate Died = Stime <= 5.0

replace Stime = 5.0 if Died == 0

gsort Treatment

gen ID=_n

//==================Conducting statistical methods===========

*a) Time-fixed method

stset Stime, failure(Died =1)

stcox i.Treatment, hr

*b) Exclusion method

preserve

replace Stime=Stime-Imtime if Treatment==1

stset Stime, failure(Died =1)

stcox i.Treatment, hr

restore

*c) Time-dependent method

preserve

expand 2 if Treatment==1

replace ID=1000+_n if _n>1000

replace Stime=Imtime if ID>1000

replace Died=0 if ID>1000

replace Treatment=0 if ID>1000

replace Imtime=0 if ID>1000

gen fup_dependent=Stime-Imtime if Treatment==1

replace fup_dependent=Stime if Treatment==0

stset fup_dependent, failure(Died=1)

stcox i.Treatment, hr

restore

*d) 0.5-year Landmark method

preserve

drop if Died==1 & Stime<=0.5

replace Treatment=0 if Imtime>1

stset Stime, failure(Died=1)

stcox i.Treatment, hr

restore

*e) 1-year Landmark method

preserve

drop if Died==1 & Stime<=1

replace Treatment=0 if Imtime>1

stset Stime, failure(Died=1)

stcox i.Treatment, hr

restore

*f) 2-year Landmark method

preserve

drop if Died==1 & Stime<=2

replace Treatment=0 if Imtime>1

stset Stime, failure(Died=1)

stcox i.Treatment, hr

restore
